# Using photovoice to understand and improve healthy lifestyles of people diagnosed with serious mental illness

**DOI:** 10.1111/jpm.12863

**Published:** 2022-08-20

**Authors:** Amanda J. Edmondson, Rachel Borthwick, Elizabeth Hughes, Mike Lucock

**Affiliations:** ^1^ Nottingham Trent University Nottingham UK; ^2^ South West Yorkshire Partnership NHS Foundation Trust Wakefield UK; ^3^ Edinburgh Napier University, School of Healthcare University of Leeds Leeds UK; ^4^ University of Huddersfield, South West Yorkshire Partnership NHS Foundation Trust Wakefield UK

**Keywords:** care planning, formulation, health research, mental health services, photovoice, qualitative

## Abstract

**What is known on the subject?:**

People diagnosed with serious mental illness (SMI):

- Live 10 to 20 years less than the general population, and this can be related to lifestyle factors such as poor diet and low levels of physical activity.
- Have a good understanding of what healthy lifestyle comprises of, but face barriers and challenges related to their mental health, treatment, and life situation.

There is limited participatory research that considers the specific beliefs of people diagnosed with SMI about what “being healthy” means to them.

**What this paper adds to existing knowledge:**

- People diagnosed with SMI value health and are often already engaged in activities that promote both physical and mental health.
- They experience the “vicious cycle” of barriers to engage in healthier lifestyle, including medication effects, poor sleep routines, fatigue, low mood and establishing a routine, but this shows how healthy activities can improve their mental health.
- The importance of meaningful places and their role in supporting healthy lifestyles was identified
- Some people diagnosed with SMI face significant socio‐economic challenges (such as lack of cooking facilities; limited money for purchasing healthy food) to support healthy lifestyles.
- To truly understand the perspectives of people with SMI, who are typically voiceless and disempowered, research methods need to allow the participants to set the agenda for discussion, to not only provide rich data but also have the added benefit of empowerment and enhanced engagement.

**What are the implications for practice?:**

Mental health nurses should:

- Explore the practical barriers to healthy lifestyle such as financial concerns and ensure that people can access support to obtain what they need from the local community resources.
- Instigate a mental health and/or medication review if mental health symptoms or medication side effects are a barrier to healthy lifestyles
- Explore what places have meaning and consider how to use meaningful places as motivating factors for healthy lifestyles and promoting mental well being.

**Abstract:**

**Introduction:**

People diagnosed with serious mental illness (SMI) live 10–20 years less than the general population, due in part to co‐existing physical illness linked to lifestyle factors. To inform individualized care plans to promote healthy lifestyles, it is important to understand the views of people diagnosed with SMI. To truly understand their lived experience, research methods should allow participants to set the agenda for discussion, enhancing engagement and empowerment in the research process.

**Aim:**

To use a participatory research approach to capture what healthy lifestyle means to people who are diagnosed with SMI.

**Method:**

Eight people diagnosed with SMI participated in six, weekly focus groups using Photovoice. Data were analysed using thematic analysis.

**Results:**

The overarching theme was ‘mental health is the main priority’, and the other themes were barriers to a healthy lifestyle, represented as a vicious cycle, and three themes, which were facilitators ‐ the importance of place, meaningful activities, and the importance of others.

**Discussion:**

The methodology allowed participants to choose images that reflected their lived experience. The themes describe the interaction of physical and mental health and practical barriers and will inform the design of individualized care plans.

**Implications for Practice:**

In co‐designing care plans, mental health nurses should draw on peoples' preferences and explore the barriers identified in this study.

## 1 INTRODUCTION

The life expectancy of people diagnosed with serious mental illness (SMI) is approximately 10 to 20 years less than the general population (Chesney et al., 2014; Laursen, 2011). SMI includes mental disorders such as schizophrenia‐spectrum disorders, bipolar disorders, and severe major depression where there is significant functional impairment and limitation of major life activities (Ruggeri et al., 2000). This increased mortality is due to high levels of co‐existing physical illness such as respiratory problems, cardiovascular disease, and obesity (De Hert et al., 2011) and lifestyle and psychiatric treatment factors account for much of the increased risk of having these illnesses. For example, people diagnosed with SMI have higher levels of obesity (Coodin, 2001) and are often more sedentary compared with the general population (Daumit et al., 2005). Furthermore, antipsychotic drugs are significantly associated with weight gain (Newcomer, 2005), metabolic syndrome (Vancampfort et al., 2015) and type 2 diabetes (Vancampfort et al., 2016).

This has led to interventions to improve the physical activity of people diagnosed with SMI, and a review (Richardson et al., 2005) noted that regular physical exercise improved mental health, especially secondary psychiatric symptoms of psychosis such as depression and low self‐esteem and that interventions that target specific groups and/or are tailored to individuals are more effective. The importance of individually tailored, or personalized, interventions is widely acknowledged in healthcare (e.g., The NHS Long Term Plan, 2019). These interventions should be informed by a good understanding of predisposing and precipitating factors contributing to the problem being addressed, and perpetuating factors that maintain the problem or are barriers to behaviour change. Individual case formulations incorporate these components and are widely used in psychological therapy and mental health services (e.g. Pearson, 2008; Tarrier & Calam, 2002). They are informed by psychological theory and more effective in planning interventions than diagnosis alone (Macneil et al., 2012). Case formulations tend to be informed by general formulations, which are then adapted to account for the unique circumstances for the individual. A better understanding of the beliefs, interests, values, and goals of individuals, what motivates them and barriers to behaviour change will support this individualized approach. More tailored approaches would also be more likely to improve self‐efficacy, the belief the individual has the capability to be able to do something successfully, which is one of the most important predictors of health behaviour change (Holloway & Watson, 2002).

There is limited research that considers the specific beliefs of people about what “being healthy” means to them. Blanner Kristiansen et al. (2015) undertook interviews with people diagnosed with SMI with the aim of understanding what they thought are the causes of physical health issues, what types of issues they experienced, and what could be done to prevent these issues. The participants regarded physical and mental health as inseparable and identified a significant impact of living with an SMI on their physical health, energy, and motivation, and the impact of medicines and dealing with symptoms of SMI. Blomqvist et al. (2018) undertook 16 qualitative interviews with people with SMI in Sweden asking “how healthy living has influenced own health, the experiences of trying to change unhealthy habits, and what helps support healthy living”. They identified a main theme of being regarded as a “whole human being” in that physical health is an important aspect of holistic care. Other themes identified included being able to get outdoors, importance of structure and planning and support from significant others. These studies offer useful insights but were based on discussions led by researchers and therefore limited to the parameters of the study. To truly understand the perspectives of people with SMI, who are typically voiceless and disempowered, participatory research methods that allow the participants to set the agenda for discussion not only provide rich data but also have the added benefit of empowerment and enhanced engagement.

A community‐based participatory research (CBPR) approach was taken using Photovoice, a method where photographs are used as an elicitation tool for facilitating discussion and sharing experiences. Caroline Wang and Mary Ann Burris developed Photovoice, a visual research methodology, which enables people to record and reflect on issues facing their community and their day to day lives (Wang & Burris, 1997). Photovoice encourages critical dialogue and knowledge about issues of concern through group discussion, with the intention to create change. Underpinning this approach is the idea that the visual image enables people to identify and think critically about problems/issues central to their lives more easily. Using a camera is also accessible and inclusive of those who do not read, write, or speak the dominant language, and those living with stigmatized health conditions, such as SMI. Photovoice is an approach, which recognizes that it is the people living in those communities who have the expertise and insight, not the professionals. Adopting such an approach has the potential to be empowering, accessible, and inclusive of those who are often passive subjects to health policy and practice (Wang & Burris, 1997). Photovoice has been used in research with people diagnosed with SMI, including Cabassa, Nicasio, and Whitley (2013) who found photovoice helped people diagnosed with SMI living in supportive housing in the US to communicate life experiences related to their health, and to formulate solutions. Their work led to the development and testing of a peer‐led healthy lifestyle program focused on weight loss, cardiorespiratory fitness (CRF), and cardiovascular disease (CVD) risk reduction (Cabassa et al., 2015). Findings indicated that peer‐led group lifestyle balance (PGLB) was not superior to usual care in helping participants achieve clinically significant changes in weight, CRF, and CVD risk reduction (Cabassa et al., 2021), which emphasizes the difficulties achieving positive outcomes for people diagnosed with SMI and the importance of identifying barriers to change. By engaging them in an exploration of the barriers and facilitators, and what a healthy life is to them, we hope the findings of this study will help us to understand factors that should be taken account of when designing personalized approaches.

## 2 AIM

The aim of the study was to work in partnership with people diagnosed with SMI to understand what healthy means to them, and the barriers and facilitators to living a healthy lifestyle. Understanding this will help the development of personalized formulations and interventions to improving healthy lifestyles of people diagnosed with SMI.

## 3 METHOD

### 3.1 Design

A ‘community‐based participatory research’ (CBPR) approach, using a focus group over 6 weeks and Photovoice, to ensure a participant‐led and lived experience perspective.

### 3.2 Sampling and recruitment of participants

The study aimed to recruit a sample of eight participants as this is the optimum number for a focus group (Stewart & Shamdasani, 2014) and consistent with previous photovoice studies (Cabassa, Parcesepe, et al., 2013). Purposive sampling was used to recruit people in receipt of care from a community mental health team (CMHT) with serious mental health conditions and equal numbers of males and females. Twelve “consent to contacts” were received of which nine subsequently consented to participate. The remaining three were ineligible for the study; two did not have a diagnosis of SMI, and the other was in hospital at the time of completing the consent to contact form. One person who consented to participate in the research subsequently withdrew because they moved out of the area. Eight adults, five males, and three females, aged between 31 and 53 years participated in the study.

### 3.3 Setting

A publicly funded NHS community mental health service in a provincial city in the North of England, UK. Life expectancy for men and women living in this city is lower than the England average (Public Health England, 2019). The wider local authority area from which participants were recruited has an Index of Multiple Deprivation (IMD) of 54 out of 317 local authorities in England (where 1 is the most deprived).

### 3.4 Recruitment

The CMHT case managers approached potentially eligible participants on their caseloads, gave leaflets about the study, and obtained “consent to contact” if the service user expressed an interest in the study. Researcher (RB) then met with potential participants to talk through the study process, and the Participant Information Sheet. Potential participants were given at least 3 days to consider their participation, before giving written informed consent.

### 3.5 Ethical and governance approval

The study was given a favourable opinion by a UK National Health Service (NHS) Ethics Committee and approved by the UK Health Research Authority. The consent form and patient information sheet made it clear that if anyone disclosed risk of harm to self or others, the researchers would have to contact emergency or mental health services as appropriate. All data were stored in compliance with the General Data Protection Act, (2018) and electronic data stored on the NHS Trust secure network drive with password protection. Data were anonymised using pseudonyms, and potentially identifiable audio‐recorded information was anonymised where necessary during transcription. Photos in which other people could be identified were not included, and this was made clear to participants.

### 3.6 Photovoice method

This method was crucial to the study in that it ensured a participant‐led enquiry based on lived experience. Photovoice uses photographs taken by participants as visual prompts to facilitate discussion to communicate aspects of their lived experiences through visual images and accompanying narratives (Minkler & Wallerstein, 2008). Rather than fitting experiences to predetermined questions, the active process of using participants' photographs encourages them to consider what is important to them (Harper, 2002; Wells et al., 2012), which is more likely to lead to an understanding of the realities of a person's life and feasible and effective interventions. The photovoice procedure and the photo‐sheet were adapted from guidance provided by Amos et al. (2012).

Six weekly group sessions were conducted, each lasting approximately 2 h. The first session familiarized participants with the aims (to generate knowledge about what health means to people diagnosed with serious mental illness) and structure of the sessions, instruction on how to use the cameras and confidentiality issues. Participants were asked to spend time throughout the following week taking (unlimited) digital photographs that in their view related to a topic chosen by the group.

Each of the subsequent sessions followed the same format. First, a one‐to one discussion with a group facilitator (AE, RB, ML, EH) to review the photos and selection of one photograph by the participant that they felt best illustrated the theme of the week. This was printed and the participant gave it a caption/title. A photo worksheet was used to help them express why their chosen photograph was important to them (see supplementary material). The second part of these sessions was the group session where participants presented and talked about their chosen photograph, and what it meant to them in relation to their health and the chosen theme. At the end of the group session, the theme for the following week's photographs was chosen by the participants. The final session focused on summarizing all the photographs presented, people's narratives and associated discussions, and encouraging participants to reflect on their participation in the project. All the focus group sessions took place in a city centre venue (not associated with statutory mental health services). The focus groups were audio recorded and transcribed.

### 3.7 Analysis

The photographs, notes taken at the 1:1 sessions and transcriptions of the focus groups were analysed using thematic analysis as described by Braun and Clarke (2006, 2019) and polytextual thematic analysis as described by Gleeson (2011, 2020). Polytextual thematic analysis acknowledges and enables more than one type of data to be analysed. It essentially follows the same key stages as a thematic analysis but moves between textual excerpts and images in the early stages of familiarization with the data and creation of basic explanatory codes. For example, each (group) transcript and set of images was scrutinized for themes in an iterative process that involved moving back and forward from text to images. Initially, textual excerpts and images were scrutinized and extracts of text that were deemed noteworthy were highlighted and qualities within the pictures were noted. The next stage was the creation of explanatory codes (a basic unit of meaning) that could be applied to the textual excerpts and images that conveyed the interpreted meaning. Following this stage, the data were managed as one source (a list of codes with their associated images and text). All coded textual data and images were then reviewed for fittingness by reviewing the text and visual data associated with each code to ensure all the data shared the same meaning. If different extracts of data or images differed in meaning, then codes were expanded (or collapsed if different codes had a shared meaning). Codes with similar properties were grouped into tentative themes, which were then refined and their boundaries demarcated by further scrutiny of the images and text that had informed the themes. Finally, each theme was defined and named.

The analysis performed by researchers (RB, AE) was data driven and inductive and discussions on the different stages of analysis involved AE, RB, and ML.

## 4 RESULTS

A total of eight people were recruited to participate in the study. On average, five participants attended each week. One participant attended session one only due to difficulties travelling to the group. Travel costs were provided; however, issues with mental health were a barrier. Despite this, the researchers (AE and/or RB) continued to make weekly telephone contact, and the participant continued to take photographs and provide written notes each week (using the photosheet provided). Twenty nine photographs were collected out of a possible 32 (eight photographs per sessions two to five).

**FIGURE 1:**
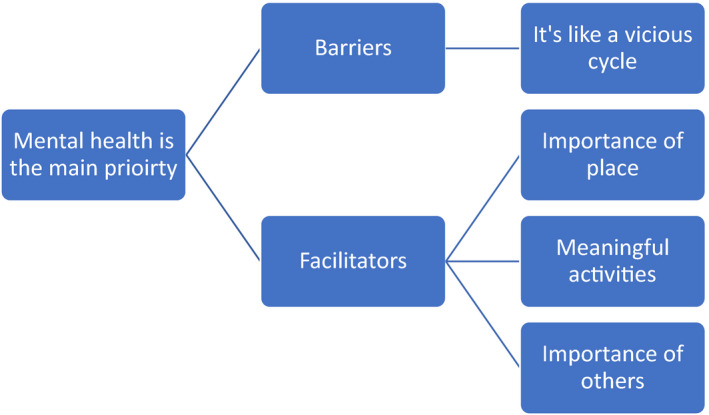
Overview of themes

Five themes were identified, which were important in terms of making healthy lifestyle choices. The first in an overarching theme, which conveys what healthy meant to people diagnosed with SMI, “Mental health is the main priority”. Barriers to a healthy lifestyle are described in theme 2, (it's like a vicious cycle), and factors that counteracted these challenges and facilitated more healthy lifestyles identified in themes 3 (importance of place), 4 (meaningful activities) and 5 (importance of others), see Figure 1.

### 4.1 Theme 1: “mental health is the main priority”

“Mental health is the main priority” underpinned all other themes. Mental health was their main focus, what mattered most to them and almost a taken for granted aspect of what healthy meant to them. Participants did not talk about physical and mental health as two separate concepts. Their main preoccupation and motivation was to be mentally well but they also recognized that many things that improve physical health (good diet, fresh air and exercise, reducing smoking and caffeine) also improved their mental health. When describing what healthy meant to them, participants described it as “feeling complete”; “feeling full”; “normal”; “being busy”; “finishing my goals”. It therefore included subjective wellbeing such as feeling “normal” and a behavioural element‐achieving goals and being occupied, which helped them feel healthy (more “capable”, “complete” and “busy”).

it's about being stable really about being you know kind of complete innit you know…it's kind of full really you know like you've got this going on here that going on there … there's loads of words I could think of like progress and being stable and being happy and things like that but to be complete to me it's about finishing my goals really (participant 7, male)

Here is an example of a personally meaningful activity for one participant:
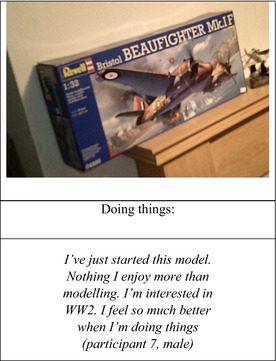


### 4.2 Theme 2: “it's like a vicious cycle”

**FIGURE 2:**
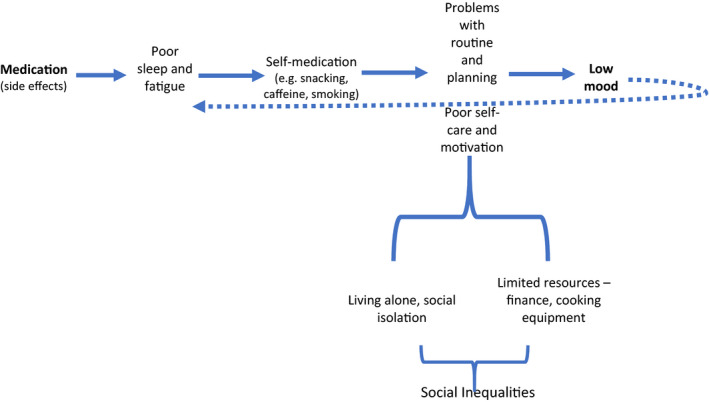
“It's like a vicious cycle”

Participants identified factors associated with living with a serious mental illness, which served as significant barriers to making healthier lifestyle choices. It was clear that these factors could at times compound each other and become a vicious cycle. An example of this, shown in Figure 2, is the experience of side effects of psychotropic medication such as weight gain, disturbed sleep, and fatigue. This, together with self‐medication such as caffeine and smoking, leads to difficulties planning, establishing a routine and self‐care, leading to difficulties engaging in meaningful activities and low mood. Social isolation and limited finances also contribute to the negative cycle.

There was a lot of discussion around medication for mental health and comorbid health issues. Participants readily acknowledged the need for psychotropic medication but also described how the side effects negatively impacted their lives in several ways, most commonly weight gain:

eating the right food on medication is hard…you get hungry, you're always wanting to eat and its awful medication sometimes, and I don't know how they haven't found a medication that doesn't actually make you feel hungry… (participant 2, female)

on medication you put weight on. I have to be careful what I eat, so that's why I'm eating salads and following traffic lights on food packaging (participant 1, male)

The medication left some participants feeling tired and two commented on needing to adapt the time they took their medication to ensure they woke on time to attend and function in the focus group (which started at 11 am). Difficulty sleeping, including waking during the night, was common. One participant described how this often resulted in drinking coffee and smoking excessively during the night, which negatively impacted on their motivation and plans for the following day:

I have a lot of trouble sleeping, I like get 2–3 h a night and I'm just up and down….I was supposed to go to meeting yesterday with [xxx] and I just stayed in…. If I keep waking up I'll have a cigarette, you're laid in bed and you know you're not going to get back to sleep so you think I may as well get up and have a cig, have a cup of tea/coffee, and it's the same thing round and round (participant 6, male)

Despite the negative aspects of medication, participants also recognized the benefits, so the challenge was finding the right balance of positive versus negative effects, but there was a reluctance to challenge professionals on their medication regimen. One participant described the challenges of taking multiple medications for coexisting mental and physical health problems, including mood fluctuations, tiredness, and confusion about how to best manage a complex prescription:
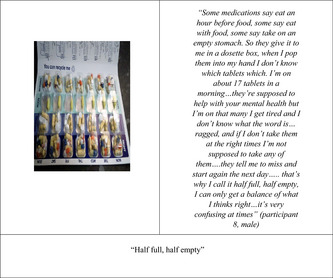


Living alone and the financial constraints that some people with serious mental illness experience also impacted on making healthy choices. One participant described cooking for one as “more difficult”, another described how fresh food is expensive and more likely to be wasted if you live alone:

I live on my own, I like to buy fresh food (but) it goes off and it's expensive to just throw stuff away (participant 3, female)

Participants described how living alone reduced their motivation to cook a meal, healthy or otherwise.

cos I live by myself you tend to cook less when you're by yourself than you are when you've got someone there, that's why I like it when my daughter comes down so I can make her somet so I'll have something along with her, so in other words she tends to help me eat cos otherwise, I wouldn't I wouldn't eat properly (participant 5, female)

Financial constraints also meant some participants were less able to cook a meal due to having no cooker:

I've never had a cooker since I moved in cos I couldn't exactly afford one; I've got a microwave and things like that but it's not the same as a cooker where you can make meals on a cooker, you can't do the same in a microwave. You're limited to what you can actually cook and I was just tending to eat junk food and that all the time (participant 5, female)

For some participants living alone, feeling less motivated to cook, and having a limited budget resulted in frequent use of fast food from cheap local takeaways:

when I'm like alright then I'll probably only have like one [takeaway] a week but there was one week where I had like four, I was just like I can't be bothered to cook and I was just too much effort”. Free delivery or 50p extra, 200 yards away – still get it delivered…don't need to leave the house (participant 3, female)

when you start totalling it up all your healthy stuff and all that what you're buying it's easier to buy fast food sometimes (participant 8, male)

Participants discussed ways of addressing and overcoming many of the issues that hindered healthier choices, and these examples could help inform tailored interventions to support health behaviour change for people diagnosed with SMI.

### 4.3 Theme 3: importance of place

The importance of “place” came up repeatedly in the discussions. Spending time in favourite places including spaces in their own home, local parks, historical sites, cathedrals, churches resulted in positive thoughts, feelings, experiences, and tapped into good memories. Most of the participants chose to visit their favourite places to take the photographs for the study. Two female participants chose home as their meaningful place; one participant took a photo of a space in her home with a reclining chair situated away from the TV. This was an indoor private space where she would go and close her eyes and relax. She described how she rarely goes out, so for her ‘healthy’ meant relaxing at home in her chair with her cat, see excerpt below:
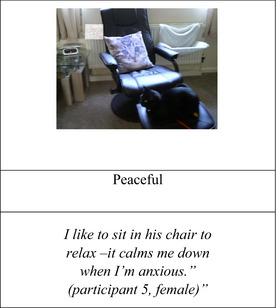


Others described visiting outdoor places because they were interesting, and/or for relaxation. For example, one participant photographed his favourite historical site and described his interest in the site, and how he perceives his interest to be healthy:

it's just my favourite place and when I think about things like that it quite interesting to think about it so I guess that's quite healthy isn't it

Another participant chose to photograph a caravan and fishery site. He described how visiting the caravan site gave him “a good health feeling”. He described eating healthier, exercising more (walking the dog), and sleeping better because of the fresh air when at the site.

### 4.4 Theme 4: meaningful activities

Participants discussed various activities, which gave them a sense of achievement and enjoyment and served to facilitate feeling healthy. For example, cooking (cooking for others, being cooked for), walking with others, or alone at night. One female participant described how walking at night was more pleasurable as it felt more leisurely and less pressured (she had nowhere to be), for her the pleasure outweighed the risks. Other activities included fishing, listening to music, drawing, exercise, and attending the weekly photovoice group.

One participant took a photograph of his own artwork alongside a speaker and described how the photograph not only captured his hobbies but also how he gauged his mental health in relation to his hobbies:
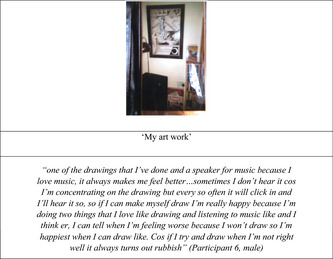


During the week when the theme was ‘food’, two of the participants brought along photographs, which captured food; however, the accompanying narratives revealed more about how the associated activity, i.e., food shopping and cooking a meal, enabled a sense of achievement and purpose:
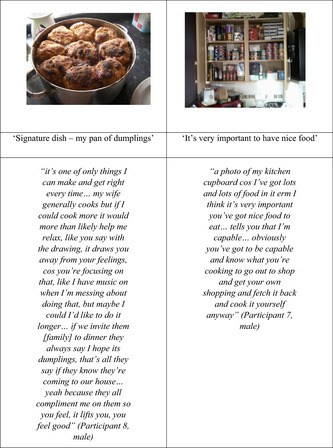


Activities involving the gym were described as difficult due to fatigue and lack of confidence by most of the participants, and one participant described how he chose to exercise at home. Where physical exercise was clearly the focus of the photograph (see below), the narrative also described how exercise helped their mental health through giving them “something to do” helping them “feel more confident and happier” and feeling more energized:
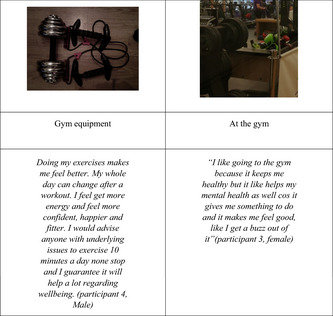


### 4.5 Theme 5: importance of others

For some participants, people (support staff, peers, family) and family pets were a key factor in facilitating a healthy lifestyle in different ways. For some, it was about having someone to do things with, such as walking the dog, going to the gym. For some, it was having someone to do things for, such as people to cook for, pets to care for. For others, it was having someone who supported and cared for them, someone to support their own ‘self‐care’ (e.g., cooking you a healthy meal). See example excerpts and images below:
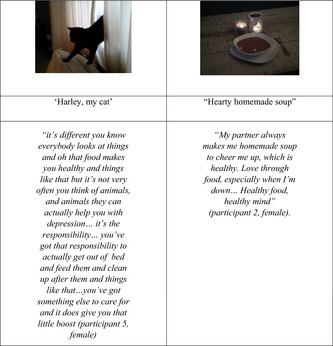


Support from mental health practitioners was identified as an important factor in overcoming a lack of confidence doing something alone, at least to get started. For example, one participant shared her story of how she wanted to join the gym but could never contemplate doing this alone. She described feeling “lucky” to receive support of her Community Psychiatric Nurse (CPN):

a lot of people said that about getting active again and a barrier being worried about like taking that first step and I think it was for me, and my CPN took me to the gym and that was like really good cos I wouldn't have ever, I wanted to go but I didn't want to go on my own and just to have like that support like not everyone has that support I think I was lucky cos my CPN was a bit of a gym bunny (participant 3, female)

## 5 DISCUSSION

There is limited research on what healthy lifestyles mean to people with SMI. Previous studies have identified that an individualized approach to health is important and that people with SMI face specific challenges that need to be overcome to adopt healthy habits. The aim of this study was to take a participatory approach to allow participants with SMI to lead and drive the discussions in relation to understanding what “healthy” means to them. A key finding, reflected in the overarching theme 1, was that the participants mainly saw “healthy” as relating to their mental well‐being, but they also informed us that many activities that focused on physical health also improved their mental well‐being (e.g., going for a walk in nature). Therefore, when promoting activities that aim to promote physical health, such as using the gym, walking, cooking groups, it may be more engaging to also acknowledge and promote the mental health benefits of these activities. In addition, people told us they are highly motivated by their own past and current interests so healthy living interventions should be flexible enough to harness individual interests, passions and capabilities, and provide support when necessary. One novel theme that emerged was the concept of feeling locked in a vicious cycle related to the interactions of the illness, medication, and lifestyle factors and how this can negatively impact on motivation, mood and self‐care. The relationship between low activity levels and low mood is well known (Elfrey & Ziegelstein, 2009), but this study highlighted additional factors that are prominent for people diagnosed with SMI, such as the effects of medication, poor sleep routines, and fatigue. The correlation between sleep problems and mental health has been established (Baglioni et al., 2011; Benca et al., 1992) and this study suggests that for people diagnosed with SMI the sedative effects of psychotropic medication and self‐medication (caffeine and smoking) may partly explain this relationship.

The participants also told us about some of the practical barriers they face in their daily life due to the socio‐economic impact of living with a long‐term mental illness. Limited finance, and skills and resources, experienced by participants, and for those diagnosed with SMI in general (Macintyre et al., 2018), such as lack of money, inadequate cooking facilities, and limited skills to cook fresh meals acted as significant barriers. These barriers reflect social inequalities (Marmot, 2010), which contribute to the poor health of people diagnosed with SMI. These factors were all barriers to planning and carrying out activities that could improve wellbeing. The relationships between these factors is represented in Figure 1, and this is an example of a general, nomothetic, formulation that can be used and adapted for individuals and to design personalized interventions.

It is important to consider how to overcome the barriers identified in this study and behaviour change approaches such as behavioural activation (Ekers et al., 2014) and implementation intentions (Gollwitzer & Sheeran, 2006) may have a role in establishing routines and sustained behaviour change. Self‐efficacy and empowerment are also important, exemplified by how the participants felt disempowered regarding actioning healthy choices, such as going to a local gym without support, or raising concerns about the side effects of their psychiatric medicines. It is widely recognized how empowerment and shared decision making should underpin personalized approaches, but some participants in our study did not experience this in the support they received from mental health services. It is possible that visual methods may support this through enabling people to identify and share their interests and passions, what is important to them and the support they need to enable a healthier lifestyle (e.g. Sitvast and Abma (2012); Russinova et al., (2018)).

Personalized plans that tap into past and present interests and passions and that give people a sense of meaning, purpose, and achievement will be more effective, and this is recognized through social prescribing approaches (Friedli & Watson, 2004; NHS England, 2014), which often includes social and peer support. There are good examples of such approaches for people diagnosed with SMI (Fancourt & Finn, 2019), and our study also highlighted the importance of ‘place’ as well as personally meaningful activities. These were situations where the participants felt more at ease, for example, green space and walking at night‐time. (Fancourt & Finn, 2019; Friedli & Watson, 2004; NHS England, 2014). Our study suggests there should be more focus on places and the settings in which activities take place, as well as types of activities. (Cabassa & Stefancic, 2019).

### 5.1 Strengths of the study

A participatory research method (Photovoice) was used to empower participants to drive the agenda regarding the specific topics to discuss. The participants worked with the researchers over six sessions not just a single time point, which allowed for relationships and trust to develop (both between the group members and the group facilitators) and for rich understandings of current lived experiences and meaningful issues to emerge.

### 5.2 Limitations of the study

It is possible that recruitment methods, where potential participants were approached by clinicians, may have resulted in selection bias, and possibly unequal opportunities for people diagnosed with SMI to express an interest in the study. However, recruitment methods did include placement of leaflets within community mental health hubs enabling people to contact the research team directly. Also, the participation information sheet highlighted the use of a digital camera and taking photos of the local environment, which may have encouraged those with more of an interest in photographs to come forward, limiting participation for those not interested in photography. However, during the project, at least two participants reported very little previous experience of taking photographs. It is possible that the inclusion of other participants may have highlighted other issues and themes but the analysis identified a high level of commonality across the participants, as well as previous studies (Cabassa, Nicasio, & Whitley, 2013; Cabassa, Parcesepe, et al., 2013; Scott & Happell, 2011) suggesting the findings have wide applicability for people diagnosed with SMI.

### 5.3 Implications for practice

Attention to physical health and healthy lifestyles as a part of mental health care is well established, but it is vital that mental health nurses recognize the additional challenges that people diagnosed with SMI face. Individually tailored interventions to promote physical health should consider the multitude of challenges that they face in trying to make healthier choices. These can include treatment factors such as medication effects and side‐effects, lack of money to access sport or buy healthy food, and low mood and lack of motivation. Mental Health Nurses are expected to undertake assessments of physical health needs (Haddad et al., 2016), but this needs to be more than a checklist and should be a holistic assessment performed in partnership with the person to understand their own motivators but also identify their specific challenges. Mental health nurses are in a key position to adopt an empowering approach where people with SMI should lead the conversation about healthy lifestyles and what would work for them given their own unique set of circumstances. Activities advertised as promoting health (such as walking groups) should also acknowledge the mental well‐being benefits as well. Mental health nurses should enquire about potential challenges to healthy living and be able to assist or mitigate these issues (such as adverse effects of medication, poor sleep routines, fatigue and difficulties with motivation, low mood, and establishing a routine). Incorporating healthy activities in their daily lives of people diagnosed with SMI should take account of their interests and passions, the priority they place on their mental health, the importance of the “place” in which healthy activities take place and the need for direct support.

## 6 RELEVANCE STATEMENT

There is a significant health inequality for people diagnosed with SMI. This study used a participatory method (Photovoice) to capture lived experience and sheds light on the challenges they face on a day‐to‐day basis in the adoption of healthy lifestyles. The findings will help inform mental health nurses how to address health promotion through individualized conversations that identify specific barriers, being able to advocate or address some of the barriers and work with peoples' interests, choices, and preferences in devising achievable and meaningful goals. It is vital that mental health nurses recognize the challenges that people diagnosed with SMI face.

## AUTHOR CONTRIBUTIONS

Conceptualisation: Mike Lucock, Elizabeth Hughes. Data collection, Rachel Borthwick, Amanda Edmondson, Mike Lucock, and Elizabeth Hughes. Formal Analysis: Amanda Edmondson, Mike Lucock, and Elizabeth Hughes. Writing/Review: Amanda Edmondson, Mike Lucock, Elizabeth Hughes, and Rachel Borthwick.

## CONFLICT OF INTEREST

The authors declare that there is no conflict of interest.

## ETHICAL APPROVAL

The study was given a favourable opinion by a UK National Health Service (NHS) Ethics Committee (reference 17/NW/0282) and approved by the UK Health Research Authority. Patient consent to publish the findings, including images, in journal articles has been obtained. The study conforms to recognized standards, e.g., Declaration of Helsinki.

## Data Availability

The data that support the findings of this study are available from the corresponding author upon reasonable request.
